# Viscoelastic Behavior of Human Lamin A Proteins in the Context of Dilated Cardiomyopathy

**DOI:** 10.1371/journal.pone.0083410

**Published:** 2013-12-30

**Authors:** Avinanda Banerjee, Vikram Rathee, Rema Krishnaswamy, Pritha Bhattacharjee, Pulak Ray, Ajay K. Sood, Kaushik Sengupta

**Affiliations:** 1 Biophysics and Structural Genomics Division, Saha Institute of Nuclear Physics, 1/AF Bidhannagar, Kolkata, West Bengal, India; 2 Department of Physics, Indian Institute of Science, Bangalore, Karnataka, India; 3 Jawaharlal Nehru Centre for Advanced Scientific Research, Jakkur Campus, Bangalore, Karnataka, India; Virginia Commonwealth University, United States of America

## Abstract

Lamins are intermediate filament proteins of type V constituting a nuclear lamina or filamentous meshwork which lines the nucleoplasmic side of the inner nuclear membrane. This protein mesh provides a supporting scaffold for the nuclear envelope and tethers interphase chromosome to the nuclear periphery. Mutations of mainly A-type lamins are found to be causative for at least 11 human diseases collectively termed as laminopathies majority of which are characterised by aberrant nuclei with altered structural rigidity, deformability and poor mechanotransduction behaviour. But the investigation of viscoelastic behavior of lamin A continues to elude the field. In order to address this problem, we hereby present the very first report on viscoelastic properties of wild type human lamin A and some of its mutants linked with Dilated cardiomyopathy (DCM) using quantitative rheological measurements. We observed a dramatic strain-softening effect on lamin A network as an outcome of the strain amplitude sweep measurements which could arise from the large compliance of the quasi-cross-links in the network or that of the lamin A rods. In addition, the drastic stiffening of the differential elastic moduli on superposition of rotational and oscillatory shear stress reflect the increase in the stiffness of the laterally associated lamin A rods. These findings present a preliminary insight into distinct biomechanical properties of wild type lamin A protein and its mutants which in turn revealed interesting differences.

## Introduction

The ‘fibrous lamina’ [1] underlying the inner nuclear membrane (INM) of the nucleus of most metazoan cells provides mechanical rigidity to the nucleus thus ensuring proper size and shape. Lamin A (LA) a type V intermediate filament protein is one of the major constituent proteins of the lamina along with lamin C (LC), lamin B1 (LB1) and lamin B2 (LB2). LA & LC are alternate splice products of the gene *LMNA* and expressed in differentiated cells only whereas LB1 & LB2 encoded by *LMNB1* & *LMNB2* genes respectively are expressed in mostly all cell types throughout the process of development [2]. The lamin protein(s) organize into distinctive mesh like structure inside the nucleus. Lamins exhibit general characteristic of an intermediate filament protein comprising of a central rod domain flanked by a short globular head at the N-terminal and a C-terminal tail domain. The central rod domain in turn consists of four coiled-coil domains (1a, 1b, 2a, 2b) interspersed with linker regions. *In vitro,* lamin assembly is triggered with the formation of dimers which elongate in a head-to-tail fashion into protofilaments at higher pH which further compact laterally to form paracrystal arrays under acidic pH [3]. Although lamins were isolated as detergent insoluble proteins being tightly associated with the nuclear envelope [4], [5] many studies have also established their localizations in a more soluble form within the nucleoplasm and nuclear matrices [6], [7], [8], [9]. The pool of A-type lamins within the nucleoplasm is more soluble than the peripheral lamin A [10]. Unpolymerized (hence soluble) lamin A and lamin C are distributed throughout the nucleoplasm at early G1 phase which subsequently get incorporated into the lamina over time forming partially interconnected network [11], [12]. A recent report by Kolb *et.al.*(2011), have shown by indirect immunofluorescence that lamin A and lamin C are partially segregated in lamina [12]. Similar pools of more soluble B-type lamins are also found in the nucleoplasm. There is a distinct difference in the mobility between the A-type and B-type lamins indicating different states of organization which also suggest their difference in the state of aggregation inside the nucleus. For instance, Goldberg *et.al.,* (2009) had shown that expression of somatic A- and B-type lamins in *Xenopus* oocyte produced distinctly different types of filaments – wavy and irregular bundles for LB2 and thick multi-layered ones for LA [13]. Furthermore, internal B-type lamins are relatively static whereas the A-type lamins are much more labile [14]. Silencing LA/C, LB1 or LB2 demonstrated the distinct compartmentalization and roles of each type [14]. Additionally, Fluorescence Resonance Energy Transfer (FRET) experiments have established that LA and LB1 can interact in a heterotypic fashion in live cells alongside with the obvious homotypic interactions [15] as had been shown earlier by biochemical assays with heterologously expressed purified proteins [16]. Thus, A, C and B-type lamins form interconnected yet distinct networks within the nuclear lamina and nucleoplasm. This could possibly be a hint that their physical behaviour as individual protein polymers might also differ. Interestingly more than 400 different *LMNA* mutations causing at least 11 human diseases, termed as laminopathies have being uncovered. Dilated Cardiomyopathy (DCM) is one such disease characterised by dilated left ventricle along with impaired systolic function which results in congestive heart and sudden death [17], [18]. To date 128 mutations in *LMNA* have been shown to produce DCM worldwide (http://www.umd.be/LMNA/). In contrast to overwhelming *LMNA* mutations only few diseases linked to the *LMNB1/B2* have been delineated so far. These mutant lamin A variants in these diseases produce phenotypes like blebs in the envelope, abnormally shaped nuclei, nuclear fragility, lamina thickening and mislocalization of nuclear pore complexes [2]. Although, lamin A and C are encoded from the same gene, identical mutations in lamin A and C show altogether different effects. Therefore, it suggests different roles for these two proteins in spite of their structural identity [19]. The explanation for lamin A being a causative of various diverse diseases may be arrived at from two alternative hypotheses: “structural hypothesis” and “gene regulation hypothesis” [20]. While the “structural hypothesis” tries to explain the anomalies in mechanical rigidity arising from the malformed lamin A network, the “gene regulation hypothesis” takes into account the role of the mutant lamin A proteins in transcription. In retrospect, pioneering studies which focussed on human lamin B1 protein in a different context, revealed networks possessing elastic stiffness which increased under tension and also exhibited resilience against shear deformations [21]. However, in a different study, lamin B1-deficient cells were shown to exhibit normal nuclear mechanics in spite of having significant numbers of blebbed nuclei [22]. The study by Lammerding *et.al.*, demonstrated that lamin A/C deficient cells had “misshapen” nuclei with reduced nuclear stiffness [22]. Several experimental methods like micropipette aspiration, cell strain, cell compression and atomic force microscopy [23], [24], [25] have probed mechanical properties of cell nuclei based on intact cells or isolated nuclei, where, contributions from the chromatin and filamentous meshwork constituting the lamina were taken into account [26], [27]. Similar AFM studies by Schäpe *et.al.*(2009), predicting the elastic modulus of lamin A in *Xenopus* oocytes [28] lacks justification as the mechanical force response described therein stems from asymmetric contributions of nuclear envelope, non-homogeneous lamin A layer and the underlying nucleoskeleton. Nuclear lamina acts as the major shock absorber of the nucleus [27]. In stiff tissues (muscle, heart, bone) lamina is dominated by lamin A filaments. Lamin A expression elevates upon experiencing high stress which shows a dominating contribution to nuclear viscosity over lamin B’s network. However, low level of lamin A in these stiff tissues provides insufficient protection to nucleus from extreme stress and may in turn lead to diseased phenotype [29]. A Recent report by Discher *et.al*., revealed lamin A as a “mechanostat” factor in cell but not B-type lamin which in turn regulate the cellular response to stress and differentiation [29]. Thus, detailed investigation of the elastic properties/stiffness of the lamin A protein and its mutants in the light of “structural hypothesis” might explain the phenotype of distorted, fragile nuclei – a major hallmark of laminopathies.

In this work, we have examined the rheological properties of lamin A protein solutions. We have showed that lamin A forms an elastic solid above a characteristic volume fraction Φ and interpreted that the transition occurs due to the formation of a network through the crossing over of lamin A rods. We further demonstrated the absence of shear hardening in the non-linear viscoelastic behaviour of lamin A protein. On probing the non-linear response of lamin A by prestressing the network with a steady shear stress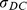, the differential elastic modulus *K′* varies linearly with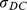, indicating a force-extension relation for lamin A where force *f* required to extend the bundle of lamin A rods diverges as 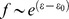 where *ε* is the extension of the bundle and 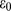 the maximum possible extension of the bundle. The major hallmark of the present study is the significantly different non-linear viscoelastic response of lamin A networks. Last but not the least; we compared the viscoelastic behaviour of two such representative lamin A mutants associated with DCM namely E161K and R190W with the wild type. These two mutants lie in the coil 1b domain of the central α-helical rod which plays an important role in the dimer formation of the lamin filaments. Mutation in this domain may lead to abnormal mechanotransduction and mechanical stress induced damage at cellular level [30], [31]. Moreover, the mutation R190W affects a highly conserved residue localized in exon 3 of *LMNA* and this exon is considered as a mutation “hot spot” in DCM [31], [32]. On the other hand the mutation E161K was reported to alter the gene expression profile in human cardiomyopathic heart [33]. The mutants discussed in this work have been reported separately in cohort of Italian, Spanish, German, American, Finnish, Irish and Korean population of DCM afflicted patients and they produce severe phenotypes resulting in sudden cardiac death [31], [32], [34], [35].

## Materials and Methods

### Expression and purification of protein

Full length human lamin A protein/pre-lamin A (664 amino acids) used for this study was expressed from pET-LA, transformed into BL21(DE3)pLysS competent cells and cultured in TB broth (Himedia, Mumbai, India) in the presence of penicillin and chloramphenicol (USB corporation, Cleveland, OH, USA); Mutants E161K and R190W were generated using pET-LA and pEGFP-LA as template by side directed mutagenesis. Primers used were E161K_sense-5'-gcacgctggagggcaagctgcatgatctg-3': E161K_antisense-5'-cagatcatgcagcttgccctccagcgtgc-3'; R190W_sense-5'-atgagatgctgcggtgggtggatgctgag-3'; R190W_antisense-5'-ctcagcatccacccaccgcagcatctcat-3. Protein expression was induced with 2 mM IPTG (Himedia, Mumbai, India) for 2 hours. Cell lysate was prepared as described by Moir *et.al.* 1991 [36] and separated on a Mono S™5/50 GL Column (GE Healthcare, Uppsala, Sweden) fractions were eluted in 6 M urea, 25 mM Tris-HCl pH 8.6, 250 mM NaCl and 1 mM DTT (Urea buffer). Proteins were renatured by dialyzing out urea in a step wise manner from 6 M in steps of two at room temperature using Slide –A- Lyzer –Minidialysis units (Thermo Scientific, Rockford, IL, USA) with a 10,000 Dalton MWCO. Assembly buffer (25 mM Tris-HCl pH 8.6, 250 mM NaCl and 1 mM DTT), has been used for all experiments with the wild type and mutant proteins. Protein concentrations were determined by standard Bradford reagent (Bio-Rad, Hercules, CA, USA) in a Perkin Elmer Luminiscence Spectrometer. Deionised water of highest purity (Resistivity18.2 MΩ.cm @ 25°C) obtained from Synergy Millipore water purification system was used for preparing the buffers.

### Indirect Immunofluorescence

HeLa cells were maintained as described previously in (Bhattacharjee *et.al.* 2013) [37]. Transfections with pEGFP-LA and mutant constructs were conducted using Lipofectamine 2000 (Invitrogen) in accordance with the manufacturer’s protocol. Transfection efficiency was routinely checked by fluorescence of GFP in an inverted fluorescence microscope. Transfection efficiencies were around 70%. For indirect immunofluorescence cells were treated as described previously in (Bhattacharjee *et.al.* 2013) [37] images of fixed cell were acquired with an LSM510 confocal microscope (Carl Zeiss) with oil immersion objective lenses (63×). Images were analysed using Axiovision version 4.8 (Carl Zeiss) and Image J software for calculation of mesh size within the lamina as described earlier by Shimi *et.al*. 2008 (See Materials and Methods) [14].

### Scanning Electron Microscopy (SEM)

Renatured protein samples assembled in assembly buffer were spotted on circular coverslips (Ф-13mm), dried in vacuum and coated with gold in IB2 Iron Coater. Samples were imaged in Hitachi S530 Scanning Electron Microscope (Japan) between 800x-3000x magnifications at 25 kV.

### Immunoblotting

The authenticity of the proteins used in SEM and other biophysical studies were confirmed by immunoblot analysis. Proteins separated on 10% SDS-PAGE were electro blotted onto 0.45 µm nitrocellulose membrane (Millipore, Temecula, CA, USA) and probed with mouse monoclonal anti Human Lamin A+C (JoL2) antibody (Millipore, Temecula, CA, USA) and Stabilized Peroxidase Conjugated secondary Goat Anti-Mouse (H+L) antibody (Thermo Scientific, Rockford, IL, USA) at dilutions of (1:200) and (1:3000) respectively. Chemiluminescence was developed on Kodak Medical X-ray Films using Super Signal West Pico Chemiluminescent Substrate (Thermo Scientific, Rockford, IL, USA).

### Circular Dichroism (CD) Spectroscopy

Far-UV CD spectra of full length human lamin A protein were recorded at 25°C in a Jasco J-720 Spectropolarimeter with a quartz cuvette having a path length of 1 mm. Proper refolding of wt LA from the denatured state (6 M urea) was established by CD spectroscopy where the spectra of 0.7 mg/ml of wild type protein were recorded from 210 nm to 250 nm (in the presence of urea) and from 200 nm to 250 nm (in the absence of urea) after extensive dialysis in 4 M, 2 M urea buffer and finally in the assembly buffer separately.

### Rheological measurements

The rheological measurements were carried out in a stress controlled rheometer (MCR 300, Anton Paar, Graz, Austria) which can also be operated in a strain-controlled mode through a feedback mechanism. The lower plate is fixed and the shear deformations were applied by rotating the upper cone (cone diameter 25 mm, cone angle 2°) in a controlled manner. Wild type and mutant proteins in assembly buffer were loaded between the cone-plate at 20°C. The protein samples were placed in a humidified chamber (with buffer solution) during the measurements, to prevent evaporation of the solvent. The measurements were adequately repeated. The schematic of the viscoelastic measurements has been outlined in Figure S1 in File S1.

### Dynamic Light Scattering (DLS)

Dynamic light scattering measurements were performed with human lamin A protein in assembly buffer at 25°C on a Zetasizer Nano S particle analyzer (Malvern Instruments, UK). A 4 mW He-Ne laser (632.8 nm) was used as the light source and the detector was placed at a fixed angle of 173°. A correlation curve was generated from the intensity autocorrelation function given by 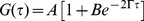, where *G* is the correlation coefficient, *A* is the amplitude of the correlation function, and *B* is the baseline;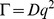, where *D* is the Stokes’ Einstein diffusion coefficient and *q* is the scattering vector. Samples were scanned for a minimum of five measurements. Cumulants analyses of the correlation curves thus generated were used to determine the intensity percentage statistics distributions, from which the number percentage distributions were derived. The mean hydrodynamic diameters at the position of maximum frequency of the number (%) distribution were obtained from the resulting curves.

## Results and Discussion

### Gel like behaviour of lamin A networks

**Figure 1:**
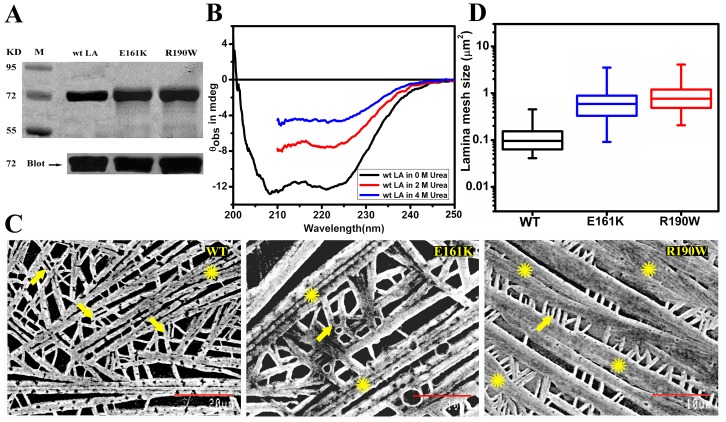
Expression, folding and ultrastructure of lamin A. A) 10% SDS PAGE analysis of pure fractions of wt LA, E161K and R190W from Mono S column; immunoblot of the same fractions using mouse monoclonal anti lamin A+C antibody (JoL2). Numbers corresponding to the bands of the marker in lane M are in kilo Daltons. B) CD spectra of 0.7 mg/ml wt LA in 4 M urea, 2 M urea and assembly buffer respectively at 25°C. C) SEM images of WT, E161K and R190W at concentrations of 0.6 mg/ml. Magnification for WT and mutants are 2000x and 3000x respectively. Scale bars for wt LA and mutants are 20 µm and 10 µm respectively. Arrow and Asterisk marks indicate the cross-linked sites and bundled filaments in the network respectively. D) Mesh size of lamina from EGFP tagged wt LA and mutants transfected in HeLa cells were calculated from confocal images and represented as box plot (n =  200–300, in 10 nuclei).

Purified full length human lamin A/pre-lamin A alternately referred to as wt LA and mutant proteins as E161K, R190W were analysed on 10% SDS-Polyacrylamide gel and blotted with monoclonal antibody (JoL-2) to show its homogeneity and authenticity in preparation respectively (Figure 1A). Proteins used for the experiments were in their properly folded state as the CD spectra of the renatured proteins in the assembly buffer had a predominance of α-helical structure (Figure 1B), characteristic of lamins. On removal of urea there was a marked increase in helicity pertaining to the gradual refolding of the proteins. Under identical conditions, similar spectral features guaranteed the proper renaturation of the mutants.

Scanning electron micrographs revealed quasi-cross-linked rod like structures as observed earlier by Goldberg *et.al.* in 2008 [13] however, no filamentous structures were observed when the cover slips coated with assembly buffer without protein were imaged. Interestingly, E161K and R190W showed an increased proportion of parallel arrays of rods compared to wt LA. Two important observations from the micrographs are the following i) similar magnification images revealed that R190W exhibited thicker bundled filaments in the network and ii) the orthogonal orientation of filaments appeared to be reduced in both the mutants compared to the wild type, with R190W exhibiting a greater tendency to form parallel arrays of rod over E161K as shown in Figure 1C. It should be emphasized in this context that these observations are in agreement with the DLS experiments published earlier [37], where the hydrodynamic diameter of R190W was shown to be higher compared to others. The reduction in criss-cross density of the networks in the case of the mutant proteins would eventually result in bigger mesh size of the lamina which was also confirmed by mesh size measurements of EGFP lamin A networks in transfected nuclei, visualized by confocal microscopy (Figure 1D and Figure S2A in File S1). Thus, the bigger mesh sizes of the lamina formed by mutant proteins in *ex vivo* condition validate our observations from SEM images in *in vitro* condition. The network formation by the wild type and mutant proteins was also established by AFM imaging. Height profile analysis from AFM imaging (Figure S3 in File S1.) also pointed to a difference in network organization between the wild type and the mutants. It should be emphasized that from here on we would adopt a convention to be followed throughout the text - time scales of seconds and hours abbreviated as s and h respectively. It must be noted that neither divalent cations like Mg^2+^ nor cross-linking proteins were added to the solutions to induce “cross-linking”.

**Figure 2:**
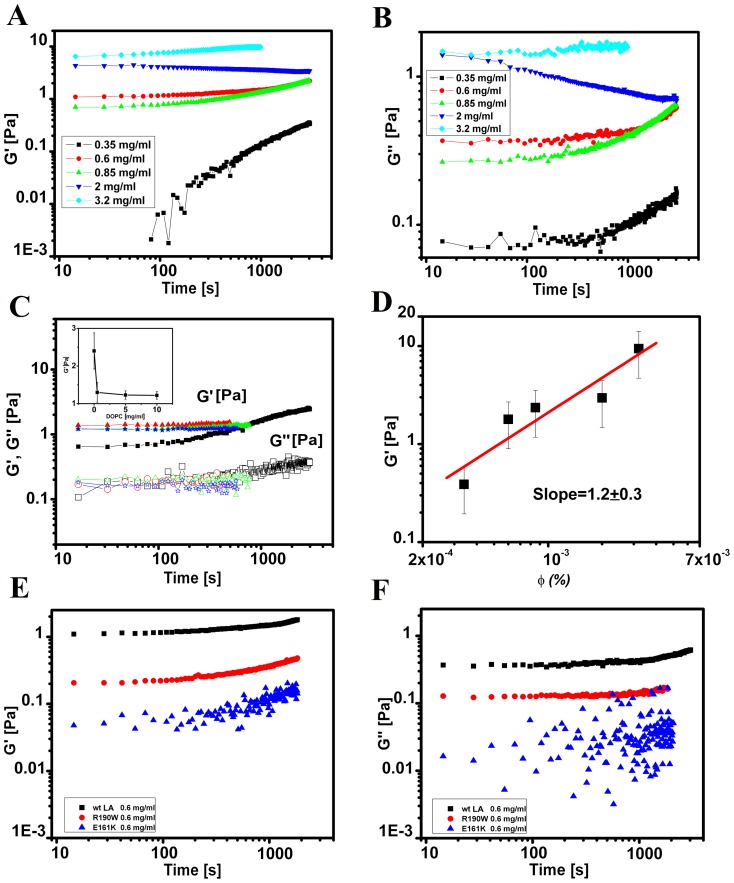
Elastic Behaviour of wt LA and mutant proteins. A) Increase in storage modulus *G′* and B) loss modulus *G′′* of wt LA upon assembly in lamin A assembly buffer with increasing concentrations. *G′* and *G′′* are the in-phase and out of phase components respectively, of an oscillatory shear of strain amplitude 1% at an angular frequency of 5 rad/s for 1000 – 3000 s. Protein concentrations used were in the range of 0.28–3.2 mg/ml of wt LA. C) Same measurement as in (A) and (B) with wt LA concentration fixed at 2.2 mg/ml and DOPC concentrations in the range 0 – 10 mg/ml. The decrease in *G′* with different DOPC concentration at the air/water interface is shown in the inset. The *G′* values obtained from repeated measurements lie within the experimental error bar. D) Concentration dependent increase in *G′* of wt LA. Comparison of E) Storage modulus *G′* and F) Loss modulus *G′′* of wild type and mutants upon assembly in assembly buffer. 0.6 mg/ml concentration of wt LA, E161K and R190W were used for these measurements. The parameters for (E, F) are identical to (A, B).

We performed oscillatory shear measurements on assembled wt LA protein solutions within a range of 0.28 – 3.2 mg/ml to determine *G′* (*ω*) and *G″* (*ω*). In principle, for viscoelastic solutions, when a sinusoidal shear deformation 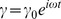 at an angular frequency of *ω* and a strain amplitude of 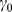
is applied, both in-phase and out of phase responses are obtained for the measured oscillatory shear stress (*σ*). The frequency dependent elastic modulus of the network *G′* (*ω*) is obtained by dividing the in-phase component of the stress by the strain amplitude. Similarly the viscous modulus *G″* (*ω*) can be obtained by dividing the out of phase component of the stress by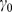. The samples were subjected to an oscillatory shear of strain amplitude 1% at an angular frequency of 5 rad/s over varying period of 1000–3000 s. Thus, we ensured that *G′* and *G′′* reached saturation before carrying out further measurements (Figure 2A, B). At 0.28 mg/ml of wt LA, the solutions sheared up to 3 h continues to remain viscous (*G′*  =  0). Hence, this has not been represented in Figure 2A. However, from the concentration of 0.34 mg/ml of wt LA, *G′* started to increase from zero on shearing for 50 s, though remaining lower than *G′′*, indicating a lingering liquid-like response at these angular frequencies. With further shearing, after about 600 s, a solid-like response set in (*G′* >*G′′*) which is the typical time for the onset of gelation at this concentration. Since, the gelation time is inversely proportional to concentration, higher concentrations of the protein (0.6 mg/ml of wt LA onwards) exhibited a solid-like behavior immediately after loading the sample. The Phase angle of lamin A networks formed under shear was plotted as a function of time (Figure S4 in File S1), where phase angle,
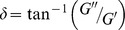
; *δ*  =  0 for solid and *δ*  =  90 for liquid.The Phase angle decreased with time at constant frequency and amplitude under oscillatory shear which could be inferred due to the formation of gel network which saturated with time also apparent from Figure 2A and B. Hence, the decrease in *G''* is definitely not due to fluidization.

Since the lamin A protein is expected to be hydrophobic as other intermediate filament proteins [38] we further verified whether the observed viscoelasticity arose from interfacial effects when the proteins accumulated at the air-water interface. As the addition of surfactants can displace the proteins from the interface, we have added different concentrations of dimyristoyl phosphatidyl choline (DOPC) dissolved in chloroform, ranging from 0.05 to 10 mg/ml at the air/water interface, as described previously [21], [38]. As shown in Figure 2C, at a wt LA concentration of 2.2 mg/ml, no significant change in the storage (*G′*) or loss moduli (*G″*) was observed from Figure 2A and B **,** indicating clearly that the measured mechanical properties of wt LA protein suspensions corresponded strictly to the bulk viscoelasticity. Figure 2D shows that the increase in *G′* with the volume fraction of lamin A rods (Φ) can be fitted to a functional form *G′* ∼ Φ^x^ where *x* = 1.2±0.3. This is in excellent agreement with the viscoelastic behaviour of semi-dilute solution of semi-flexible polymers [21], [39]. Furthermore, the mutants E161K and R190W exhibited lower *G′* and *G′′* values compared to the wild type (Figure 2E and F). This may arise from the relatively lower cross-link density and increased bundling in network compared to wt LA as observed from SEM images (Figure 1C). This would facilitate the sliding past motion of the mutant filaments on application of shear. Both the mutants represented similar trends over the range of the concentrations probed. So, we focused on 0.6 mg/ml as a representative concentration for comparison of the buildup profile of the mutants E161K and R190W with that of the wild type (Figure 2E and F). Therefore, from this part, it can be concluded that lamin A in solution, behaves as a suspension of quasi-cross-linked rods whose contour length might be comparable to the persistence length and mutant proteins exhibit distinctly different network formation ability from wild type.

**Figure 3:**
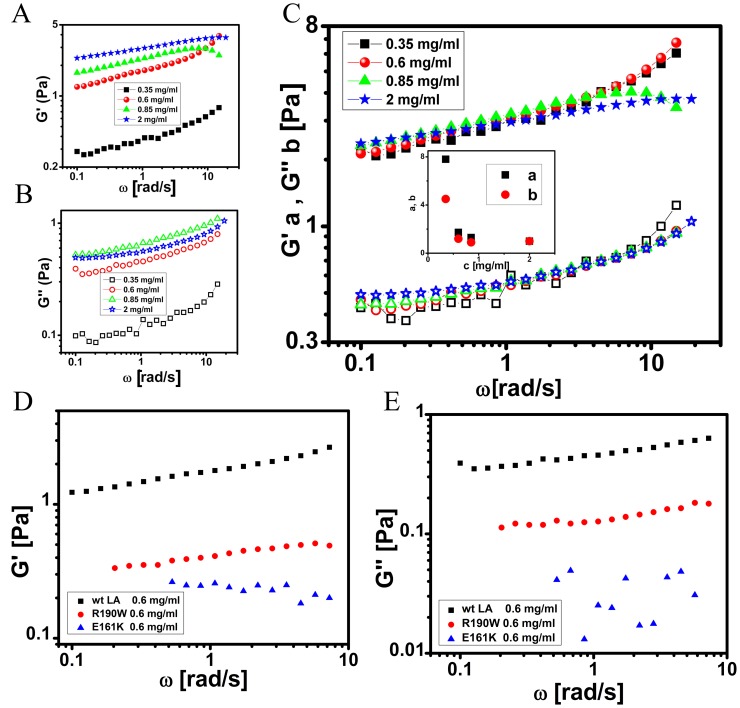
Frequency sweep measurements of wt LA and mutant proteins. Measurements for A) elastic modulus *G′* (*ω*) and B) viscous modulus *G′′* (*ω*) were carried out for probing the structural relaxation in the gel phase by varying the angular frequency in the range 0.1 to 20 rad/s with the strain amplitude fixed at 1%. C) Master curve of the linear viscoelasticity of the lamin A network. Protein concentrations used were in the range of 0.28–2 mg/ml. The variation of the scaling parameters for *G′* and *G′′* are shown in the inset where **a**- *G′* and **b**- *G′*′. Measurements for D) elastic modulus *G′* (*ω*) and E) viscous modulus *G′′* (*ω*) of wt LA, E161K and R190W at 0.6 mg/ml concentration were carried out for probing the structural relaxation in the gel phase by varying the angular frequency in the range 0.1 to 10 rad/s with the strain amplitude fixed at 1%.

Subsequently, frequency sweep measurements were performed to probe the structural relaxation in the gel phase by varying the angular frequency in the range 0.1 to 20 rad/s with the strain amplitude fixed at 1% (Figure 3A and B) which corresponds to the linear viscoelastic region. The viscoelastic spectra obtained indicate that *G′* >*G′′* at the frequencies probed. The absence of structural relaxation and the weak frequency dependence of the storage and loss moduli suggest that the rods of lamin A might criss-cross to give a quasi-cross-linked appearance of gel in solution above a critical concentration corresponding to the sol-gel transition. It must be borne in mind that there is no physical covalent bonding at the sites of the cross-links. Moreover, for concentrations in the range of 0.34 mg/ml to 2 mg/ml of wt LA corresponding to the gel phase, the viscoelastic spectra could be scaled on to a master curve (Figure 3C) suggesting that the structure of the network remained independent of the concentration in the gel phase [40]. E161K and R190W show similar kind of relationship between structural relaxation and angular frequency of storage and loss moduli with characteristically low *G′* and *G″* (Figure 3D, E). Thus, we observed nearly frequency independent viscoelastic spectra with *G′* >*G′′* over the range of frequencies probed in either of cases of wild type and mutants, indicating a large structural relaxation time for the suspension; a behaviour distinct from that observed for a suspension of rods above the overlap concentration [41]. Therefore, it could be concluded from linear viscoelastic measurements that lamin A protein undergoes a sol to gel transition from 0.35 mg/ml onwards. With a typical rod diameter D ∼ 10 nm and length L ∼ 50 nm, the average aspect ratio of the lamin A rods is ∼ 5. Hence, the volume fraction Φ corresponding to the overlap of rods in solution occurs at 24 (D/L)^2^ [42] signifying a transition from dilute to semi-dilute region at Φ_t_ ∼ 0.96. Typically, the rheological sol to gel transition occurs at Φ_t_ =  0.0003 (corresponds to *c*  =  0.3 mg/ml), which is orders of magnitude lower than the overlap concentration of lamin A rods estimated above; suggesting probably that the elastic behaviour might arise from the cross-linking of lamin A rods. This is because the non-covalent hydrophobic interactions result to quasi-cross-linking, thus preventing a thermally induced structural relaxation over the time scales probed.

### Non-linear elasticity of lamin-A networks

**Figure 4:**
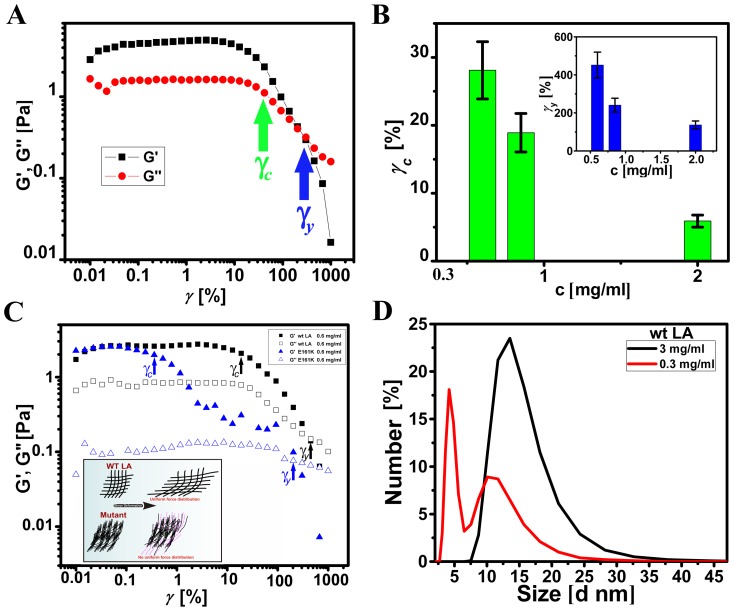
Strain induced changes in the network of wt LA and mutant protein and DLS measurements. A) Dependence of elastic modulus *G′* and viscous modulus *G′′* of wt LA at 0.85 mg/ml concentration on varying the strain amplitude 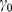 in the range of 0.01 to 1000%, keeping the angular frequency fixed at 5 rad/s. Green and blue arrow indicates critical strain 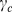 and yield strain 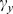 respectively. B) Concentration dependence of the critical strain 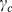 corresponding to the onset of non-linearity and the yield strain 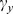
(inset) above which the network starts to flow is shown. Concentrations of 0.6, 0.85 and 2 mg/ml were used for this experiment. C) Dependence of elastic modulus *G′* and viscous modulus *G′′* of wt LA and E161K at 0.6 mg/ml concentration on varying the strain amplitude 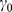 in the range of 0.01 to 1000%, keeping the angular frequency fixed at 5 rad/s. Black and blue arrows indicate critical strain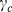, grey and light blue arrows indicate yield strain 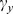 of wt LA and E161K respectively. Inset shows a model representing the fate of wt LA and mutant LA network upon shear deformation. D) Number percentage statistics of 0.3 and 3 mg/ml of wt LA protein.

To study the strength of the cross-linked network, an oscillatory shear of varying strain amplitude 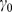 in the range of 0.01 to 1000% was applied to the wt LA protein at a concentration of 0.85 mg/ml, keeping the angular frequency fixed at 5 rad/s (Figure 4A). At low strain amplitudes, typically both the elastic and viscous moduli remained independent of strain amplitude, which corresponds to the linear viscoelastic regime. Theoretically, at higher strain amplitudes above a critical value that is sample dependent, *G′* (*ω*) can increase or decrease with the imposed strain. The former is referred to as strain hardening/strain stiffening and the latter as strain thinning behaviour. In this present study with wt LA, *G′* and *G′′* remained nearly constant for 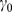
< 10%, which indicated the linear viscoelastic regime. With further increase in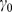, the decrease in *G′* and *G′′* revealed a monotonic shear-thinning behaviour. It should be noted that the viscoelastic response though non-linear, remained solid-like up to large strain amplitude (>100%), above which the network yielded. The critical strain 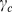 at which the network softened and the non-linearity set in, as well as the yield strain 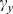 corresponding to which the network transformed from an elastic to viscous behaviour, decreased with increasing lamin A concentration (Figure 4B). In amplitude sweep measurements, the shear thinning behaviour of the complex viscosity confirmed the softening of the network even at low strain values (∼20%). It is very likely that the softening might occur through the relaxation of cross-links under the imposed strain. Based on these results we can model the loosely positioned near orthogonal cross-link contact points giving way to longitudinal orientation of the lamin A rods, along the direction of the shear, a feature which is different from that of other biopolymer networks including lamin B1 [21]. This may lead to the non-affine motion of the cross-link points and the rods without interfering with each other, thus reducing the overall stress [43]. The yielding of the network which occurs at higher strain values (> 100%) further indicates that some cross-links are indeed retained in the network up to the yield point. Interestingly, as shown in Figure 4A though this non-linearity of the lamin-A network (indicated by the decrease in *G*′) set in at a critical strain amplitude 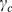 of 20%, the rupture of the network (leading to *G′* < *G′′*) occurred at a strain 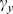 of 500% which is an order of magnitude higher than that observed for actin filaments at similar concentrations [44], though comparable to lamin B1 networks [21]. However, in case of E161K the non-linearity set in at 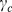
of 0.4%, while the rupture of the network occurred very early at 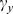 of 193%, thereby pointing to a relatively loose network with high entropic fluctuation (Figure 4C). It is equally relevant to note that 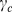 and 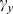 for wt LA network is concentration dependent (Figure 4B). In addition, it is interesting to note that large strain deformation amplitude (leading to a shear thinning behaviour) can lead to orientational ordering of the stiff lamin A rods resulting in bundles thus changing the morphology of the network. The absence of strain stiffening for lamin A and its mutant as compared to other biopolymer networks suggests that the lamin A proteins intrinsically do not exhibit entropic elasticity [45]. Therefore, it can be conjectured that increasing the concentration of lamin A leads to the formation of laterally aligned rods favourably over transverse alignment. Our hypothesis is supported by the findings of Goldberg *et.al.* [13] where somatic lamin A over expression in *Xenopus* oocyte has been shown to form tight bundles and an overall multi layered sheet like structure compared to the cross connected B-type lamin. We performed dynamic light scattering experiments to seek support in favour of our hypothesis.

The correlation function obtained from DLS measurements can be used to generate number percentage distribution via the intensity profile. This gives a direct correlation of the number of particles present in the solution to its size. In this study, we observed significant alteration in the profile (Figure 4D) of the number percentage distribution against size (nm) for two distinct wt LA concentrations of 0.3 mg/ml and 3 mg/ml, the range of amplitude sweep measurement. Analyses of the profiles indicate an increase of size from 10.4 nm in the case of 0.3 mg/ml to 13.54 nm for 3 mg/ml solution. This could be an effect of the bundling of the filaments. Furthermore, we observed a more homogeneous population at higher concentration with increased diameter which might correspond to an oblate spheroid shape closely resembling a predominantly bundled structure. On the other hand the size distribution at low concentration might arise from an averaged contribution of a loosely attached, quasi-cross-linked, entropically fluctuating structure and laterally bundled lamin A rods. Therefore, we conclude that bundling increased favourably at higher concentration.

**Figure 5:**
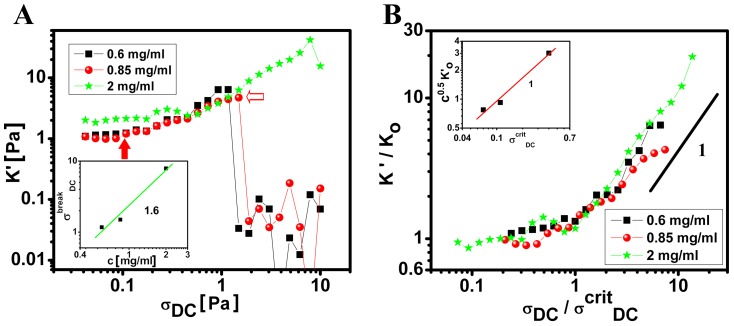
Differential elastic modulus measured from forced oscillations about a prestress. A) The differential elastic modulus (*K′*) as a function of steady shear stress (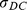) at 0.6, 0.85 and 2 mg/ml concentrations of wt LA protein. The inset shows the variation of maximum stress for breaking the network (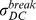) with concentration (c). Open and Solid arrow indicates 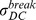 and 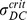 respectively. B) *K′* scaled by differential modulus in the linear region (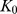) as a function of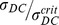. The inset shows *K′* scaled by concentration as a function of
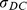
_._

In addition to a large amplitude oscillatory shear, the non-linearity of the network was quantified through a forced oscillation about a prestress [46]. This was carried out by imposing an oscillatory shear stress of amplitude 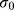 at an angular frequency *ω* on a steady rotational shear stress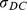. This differential measurement applied to a nonlinear state helps us to understand the origin of the elasticity of the network. We have determined the differential elastic modulus 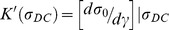 as a function of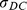, at different concentrations of wt LA in the gel phase (Figure 5A). By examining the strain values (corresponding shear rates) at these imposed stresses, we ensured that no irreversible flow occurs in the suspension at these stress values. The differential modulus remained independent of applied *DC* stress, and is same as the low frequency plateau of *G′* measured in frequency sweep experiments. This was valid only up to a critical stress value 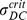 (which at 0.6 mg/ml is 0.3 Pa). Interestingly, above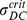, *K*′ increased linearly with 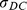 for all the concentrations under study, as evident from Figure 5B inset, 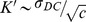. At higher values of stresses corresponding to 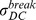, the network snapped drastically. This maximum value of stress had a concentration dependence where 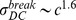 (Figure 5A, inset). At the stress 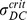 where *K′* started to increase, the measured strain was ∼ 20% (Figure 5A) corresponding to the onset of strain softening in amplitude sweep measurements (Figure 4A). This indicates that the measured differential modulus *K′* corresponded to that of the strain softened network. Further, as the breaking point of the network was reached (Figure 5A); imposed strain exceeded 500% corresponding to 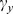 (Figure 4A). The increase in overall stiffness *K′* of the prestressed network could occur from the stretching of the cross-linked bundle formed by lamin A rods oriented in the direction of the shear. It is to be emphasized that the increase in *K′* was observed only for wt LA concentrations > 0.45 mg/ml (much above the sol to gel transition) (Figure 2D).

We therefore propose that the force *f* required to stretch the bundle of lamin A rods diverged as 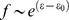 where *ε* is the extension of the bundle and 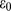 the maximum possible extension of the bundle, from which it follows that 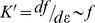, consistent with the observed increase of 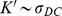 (Figure 5B). Thus, it is relevant to point here that the differential elastic modulus measured presently was likely to be that of the bundled lamin A rods rather than that of single filaments, which gives rise to a force-extension relation for lamin A that is different from other biopolymer networks (showing 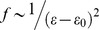) [39], [40], [47]. The unique aspect of the present study is an apparently conflicting non-linear response where *G′* decreased in non-linear regime, though *K′* showed an increase up to a critical stress. The non-linear response of the network scales as 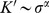, and for rigid cross-linked network of rods the scaling of *α* value was 1.5; and dense flexible cross-link networks showed scaling with slope of *α* value 1 [47], [48]. In our systems as shown in Figure 5B *α*-value of 1 corresponded to flexible weak network bonds which could break under mechanical stress and also has been seen in other filament networks [49].

### Flow Behaviour of lamin A networks under steady shear

**Figure 6:**
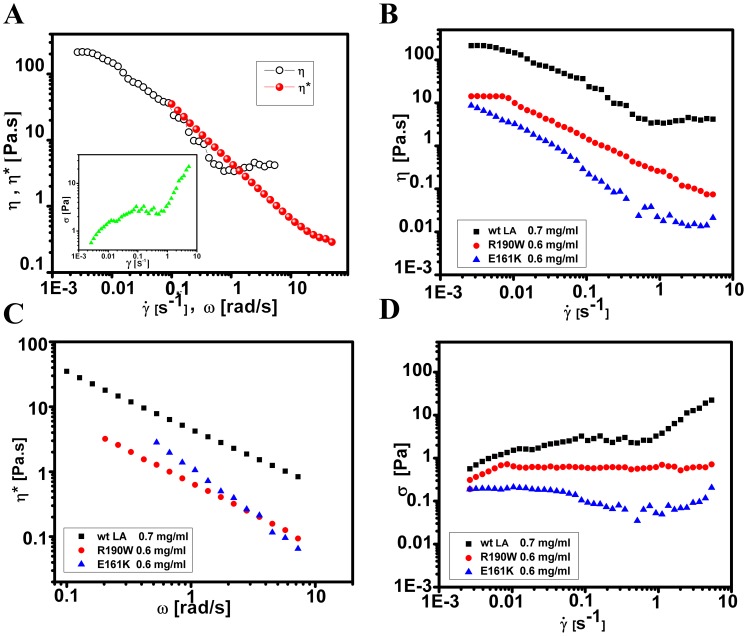
Viscosity of wild type and mutant lamin A networks under shear. A) Steady shear viscosity (*η*) as a function of shear-rate denoted by open circles and complex viscosity (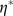
(ω)) as a function of angular frequency (*ω*) denoted by solid circles are shown. The inset shows the flow curve of wt LA (0.7 mg/ml) indicating the variation of shear stress (*σ*) with shear-rate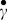. B) Steady shear viscosity (*η*) as a function of shear-rate and C) complex viscosity (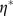
(ω)) as a function of angular frequency (*ω*) of 0.7 mg/ml of wt LA and 0.6 mg/ml of E161K and R190W. D) Flow curve indicating the variation of shear stress (*σ*) with shear-rate 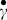
of 0.7 mg/ml of wt LA and 0.6 mg/ml of E161K and R190W.

These measurements were carried out by applying a steady torque that leads to a continuous rotation of the upper cone. To study the flow behaviour of the samples, the samples were subjected to a rotational shear stress which was varied logarithmically in a controlled manner, typically in the range of 0.001 to 10 Pa, with a waiting time of 10 s for each data point and the corresponding shear rates were recorded. In another experiment, the flow curves were also obtained by subjecting the protein samples to different shear rates (waiting time of 10 s for each data point); typically in the range of 0.01 – 100 s^−1^, and measuring the corresponding shear stress. The flow behaviour of wt LA networks under steady shear was examined (Figure 6A). Since the flow curves remained similar over the range of wt LA concentrations studied, a typical flow curve of wt LA at a concentration of 0.7 mg/ml is shown in Figure 6A, inset. The linear region observed at very low shear rates corresponded to the local irreversible rearrangement of the networks at very low deformations. The plateau region in the flow curve signified the onset of flow. Further, the stress corresponding to the plateau in the flow curve (∼2 Pa) was higher than the breaking stress 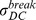 observed in the differential measurement discussed earlier (Figure 5A) confirming that no significant flow occurs below
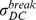
_._ Under flow, the viscosity *(η)* vs 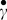
curve showed a steady shear thinning behaviour, followed by a low viscosity plateau. We have also examined whether the empirical Cox-Merz rule [50] holds for the suspension under study. Cox-Merz rule states that for a shear thinning fluid, the steady shear viscosity can be related to the dynamic viscosity obtained from linear viscoelastic measurements such that 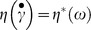 with 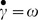. For lamin A network, the steady shear viscosity was seen to deviate from the dynamic viscosity at 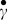
> 0.3 s^−1^. This indicates that the network structure was significantly modified from the equilibrium structure, in a steady shear flow. The observed shear thinning can thus be attributed to a shear-induced break up or “unjamming” of the gel structure of lamin A filaments under flow. The point of deviation of the steady state viscosity from the dynamic viscosity was also found to be 0.3 s^−1^ for E161K whereas R190W showed an increasingly faster unjamming behaviour (Figure 6B, C and D). It may be predicted that ionic interaction between the charged residues might play a stabilizing role in the “sliding past” movement of the rod like structures over themselves. On the other hand introduction of aromatic residues like tryptophan did not have stabilizing effect thereby expediting the avalanche in the break up or unjamming process.

## Conclusions

**Figure 7:**
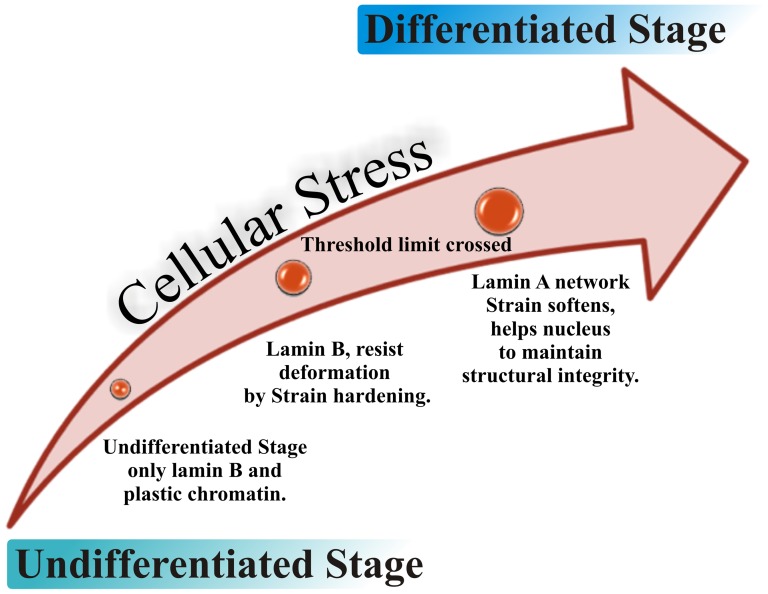
Lamin A in lamina acts as a “Check valve” in response to stress. Inside lamina A and B-type lamins respond differently with increasing cellular stress at different stages of differentiation.

This report on viscoelasticity of a nuclear intermediate filament protein is second of its kind only after lamin B1 [21]. Previously, extensive studies have been devoted to understand the viscoelastic behaviour of actin [47], [51], [52], [53] and cytoplasmic intermediate filament proteins like vimentin, desmin and keratin [38], [39], [54], [55], [56]. The major cohesion of these studies was strain stiffening behaviour of the protein polymers as an outcome of strain amplitude sweep experiments. However, one cannot extrapolate the viscoelastic behaviour of the cytoplasmic intermediate filaments to lamin A because of their different localization in the cellular milieu and hence exhibiting different functions. Interestingly, strain hardening has also been reported for cross-linked network of lamin B1 [21]. In this report we have shown that lamin A formed an elastic solid above a characteristic volume fraction Φ. Lamin A does indeed emulate the behaviour of the above mentioned examples to a certain degree. Moreover, the concentration dependence of the elastic modulus for lamin A networks was very similar to that observed for lamin B1 [21] or cytoplasmic intermediate filaments which behave like a semi flexible polymer network [38]. But the strain softening behaviour of lamin A network at large strain amplitudes was unique and strikingly different from other studied intermediate filament proteins in mammalian system. Although, a bacterially expressed intermediate filament protein Crescentin was reported to show similar strain softening behaviour which bear a 40% sequence similarity with lamin A [57]. Strain hardening essentially arises due to the low compliance of the filament when with increasing strain the average length of the filament determined by entropic fluctuations approaches its equilibrium contour length [45]. In this stretched state, the low compliance of the filament gives rise to the remarkable increase in elastic stiffness with virtually no change in strain. This has been mainly observed for cross-linked networks of actin, microtubules and intermediate filaments which form components of the cytoskeleton. In the present study, the observed strain-softening of lamin A network in strain amplitude sweep measurements could arise either from the large compliance of the quasi-cross-links in the network or that of the lamin A rods. We cannot attribute this apparent anomaly and dissimilar viscoelastic behaviour of lamin A compared to the lamin B1 to any specific reason at this time but a glance at a few notes of comparison might provide a hint as to why there is at all difference. Though all the human lamin proteins have a basic structural architecture, numerical analysis demonstrates only 53% homology between lamin A and B1 and more specifically 61% identities in the rod domain of the A and B1 [12]. A- and B-type lamins are also differentially regulated at the level of posttranslational modification. All the lamins except lamin C possess a conserved CAAX box at the carboxy terminal which is the site of attachment of the farnesyl group (C15 group) [58]. But A- and B-type lamins meet different fates with the retention of the farnesyl group. In lamin A, a second cleavage occurs at a site upstream of the CAAX box which results in the loss of the farnesyl group which is not the case of lamin B where the farnesyl moiety is retained. This suggests that the B-type lamins are more closely associated with the inner nuclear membrane compared to the A-type. B-type lamins are expressed in a constitutive manner in most or all embryonic and somatic cells whereas A-type is expressed in differentiated cells. The B-type lamins are expressed in mostly all lower metazoans [59] whereas higher organisms express both types. Barring one recent report which showed the lack of requirement of lamins including B-types in Embryonic Stem Cell development and differentiation [60] all previous studies had established the presence of B-type lamins to constitute the primordial intermediate filament network of the nucleus at the early stage of embryogenesis. Interestingly, the size of the nucleus decreases from embryonic stem cells to differentiated cells [61]. It has been also shown that human embryonic stem cells lacking lamin A/C are highly deformable and get stiffened by approximately 6-fold at differentiation with an intermediate stiffness for adult stem cell similarly lacking lamin A/C [62]. Likewise, differentiation of myoblast to myotube showed that the nuclei in the differentiated myotube stage experience stronger force and exhibit decreasing nuclear size [63]. Therefore, lamin B1 forms and retains stiff, stress resistant and porous networks [64] inside the nucleus which is reinforced by bundled lamin A filaments on top of it thus imparting necessary and adequate mechanical rigidity to the lamina [65]. This “loose” entropically fluctuating structure of lamin A bundles gets gradually aligned into parallel arrays of rods on experiencing increasing stress in the transition of nucleus with high plasticity at embryogenesis to a state of high elasticity at differentiated stage. Moreover, lamin A being present in the lamina as well as in the nucleoplasm contributes to the viscoelastic behaviour of the nucleus in a complicated manner which cannot be explained fully by power law rheology. To date, we don’t quite understand the control mechanism of elasticity of the nucleus with the combined contribution of A-type and B-type lamins in coherence with the inherent chromatin plasticity. It could be hypothesized that the primordial B-type lamins which include lamin B1 experience the forces from mechanical cues from the onset of embryogenesis and correspondingly show a progressive strain hardening behaviour up to a certain extent. These results in the stiffening of the nucleus associated with diminishing size when differentiation sets in. Thereafter, lamin A gets expressed by the completion of differentiation and eventually forms different order structures in nucleoplasm and lamina, which then acts as a “check valve” mechanism in restraining the nuclear stiffening process beyond a certain limit in response to the stress. This concept can be extrapolated to the situation when nucleus is experiencing any shear deformation during normal physiological condition. At first, B-type lamins resist the deformation showing strain hardening effect but when the threshold limit is crossed, the lamin A network softens and act as highly viscous fluid which may in turn help the nucleus to maintain its structural integrity by realigning their rods and distributing the force uniformly all over the nuclear surface (Figure 7). Again, numerous mutations in *LMNA* gene lead to different types of myopathies or muscle disorders in which muscle fibres do not stretch and relax normally resulting into different symptomatic diseases. DCM is one such myopathy where mutations in *LMNA* are major causative factors. Drawing cues from abnormally elongated and deeply indented nuclear morphology of the cardiomyocytes in *Lmna ^H222P/H222P^* mice [66] and from our recent publication [37], where we have shown structural perturbations in lamin A protein as a sequel to point mutations E161K and R190W. We reasoned that similar changes in the secondary and tertiary structure of the mutants would likewise modulate their viscoelastic behaviour. Therefore, unravelling any possible differences in the viscoelastic behaviour of the mutants with the wild type protein became an important added offshoot of this work. Present study on viscoelastic behaviour of the wt LA and mutant proteins revealed the following differences: i) network formation is significantly different in the case of the mutants; ii) this translates into a distinct difference in their elasticity and response to the shear deformation. We assume that a nucleus expressing the mutant proteins, on experiencing shear deformation will be unable to distribute the force uniformly, ultimately resulting in distortion of the nucleus as represented graphically in Figure 4C inset. Mutants studied in the current scenario are heterozygous in origin. This dictates a theoretical distribution of 50% wild type and 50% mutant lamin A proteins inside the cell. Presumably, the nuclear lamina should also depict a similar distribution. In order to mimic the heterozygous state with respect to lamin proteins inside the cell, we assembled wt LA and mutant E161K proteins in equal stoichiometry in the assembly buffer as mentioned in the materials and methods section, and visualized the network formation by SEM. SEM images revealed a network consisting more of bundled filaments over cross-linked filaments as shown in Figure S2B in File S1. Thus, wild type lamin A alone would form a uniformly cross-linked network (Figure 1C), whereas the contribution of the mutant would show a greater preponderance of parallel array of rods (FigureS2B in File S1) in the lamina of the diseased nuclei as well, thereby tipping the scale in favour of a structure containing more of bundled filaments. Therefore, we can speculate from our observation in Figure 4B, that a probable increase in 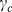 and 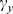, resulting from the reduction of wild type lamin A levels in DCM patients might be compensated by a proportional decrease in these values due to the contribution of increased bundling propensity of the mutant proteins. Thus, it results in yielding of the diseased nuclei even at lower strain. So, we hypothesized that these mutations would result in a relatively fragile, less elastic network which would eventually collapse even under small shear deformation ultimately exhibiting the diseased phenotype (Figure 4C inset). It must be also borne in mind that it is the interactome of the lamin A protein which eventually dictates the behaviour and function of the network in the cellular milieu. This work is the first clue as to how the effect of the mutations might perturb the elasticity of the nuclear lamina and the nucleus as such. As lamin A is involved is mechanotransduction inside the cell [29], [67], we presume that these fragile networks in unison with other nuclear proteins might be a major contributing factor in altering the elasticity of the myocytes lining the endomyocardium of the ventricular wall thus leading to ventricular dilation in DCM.

## Supporting Information

File S1 **Figure S1-S4.** Figure S1. Flow chart representing the scheme of the rheological measurements (File S1). Figure S2. Ultrastructure of lamin A network (File S1). Figure S3. Roughness profile of the *in vitro* assembled wild type and mutant proteins (File S1). Figure S4. Decrease in Phase angle *δ* with time (File S1).
(DOCX)
