# Supplementary material for: Viscoelastic Behavior of Human Lamin A Proteins in the Context of Dilated Cardiomyopathy
Source: PLoS One. 2013 Dec 30;8(12):e83410. doi: 10.1371/journal.pone.0083410 (PMC3875444; doi:10.1371/journal.pone.0083410)
Supplement: File S1 — Figure S1-S4. Figure S1. Flow chart representing the scheme of the rheological measurements (File S1). Figure S2. Ultrastructure of lamin A network (File S1). Figure S3. Roughness profile of the in vitro assembled wild type and mutant proteins (File S1). Figure S4. Decrease in Phase angle δ with time (File S1). (DOCX) [file pone.0083410.s001.docx]

**Supporting Information for** **“Viscoelastic Behavior of Human Lamin A Proteins in the Context of Dilated Cardiomyopathy”**

Avinanda Banerjee^*1^, Vikram Rathee^*2^, Rema Krishnaswamy^3^, Pritha Bhattacharjee^1^, Pulak Ray^1^, Ajay. K. Sood^2^, Kaushik Sengupta^1^

* Authors contributed equally

1. Biophysics and Structural Genomics Division, Saha Institute of Nuclear Physics, 1/AF Bidhannagar, Kolkata - 700064, West Bengal, India
2. Department of Physics, Indian Institute of Science, Bangalore -560012, Karnataka, India
3. Jawaharlal Nehru Centre for Advanced Scientific Research, Jakkur Campus, Bangalore- 560 064, Karnataka, India

To whom correspondence should be addressed: Kaushik Sengupta, Biophysics and Structural Genomics Division, Saha Institute of Nuclear Physics, 1/AF Bidhannagar, Kolkata-700064, West Bengal, India. Tel: +91-33-2337 5345 ext. 3506, Email: [kaushik.sengupta@saha.ac.in](mailto:kaushik.sengupta@saha.ac.in)

**METHOD S1**

***Atomic Force Microscopy (AFM)-*** Renatured wt LA and mutant proteins were diluted with assembly buffer containing 0.2% Gultaraldehyde to 0.2 mg/ml. After 5 min fixation at room temperature, 20-30 μl of sample were placed on freshly cleaved mica and adsorbed for 30 min, rinsed 3 times with double distilled water and air dried. AFM images were obtained with MMSPM NanoScope IV (Veeco) in tapping mode. For imaging in air, Si_3_N_4_ cantilever (RTESP7, Veeco) with a radius of 10 nm was used. Image processing was carried out using Nanotec Electronica SL WSxM software (version 5.0 Develop 6.4)

**RESULT S1**

*In vitro* assembled network of wt LA and mutant proteins at 0.2 mg/ml concentration were imaged by AFM in tapping mode. The filaments showed a height of ~3.31 nm, 1.34 nm and 1.90 nm for wt LA, E161K and R190W respectively as shown in Figure S3, which is comparable to the height observed for other intermediate filaments (vimentin) [1] when imaged in air by tapping mode. This data further proves the authenticity of the proteins as intermediate filaments used in biophysical measurements.

**
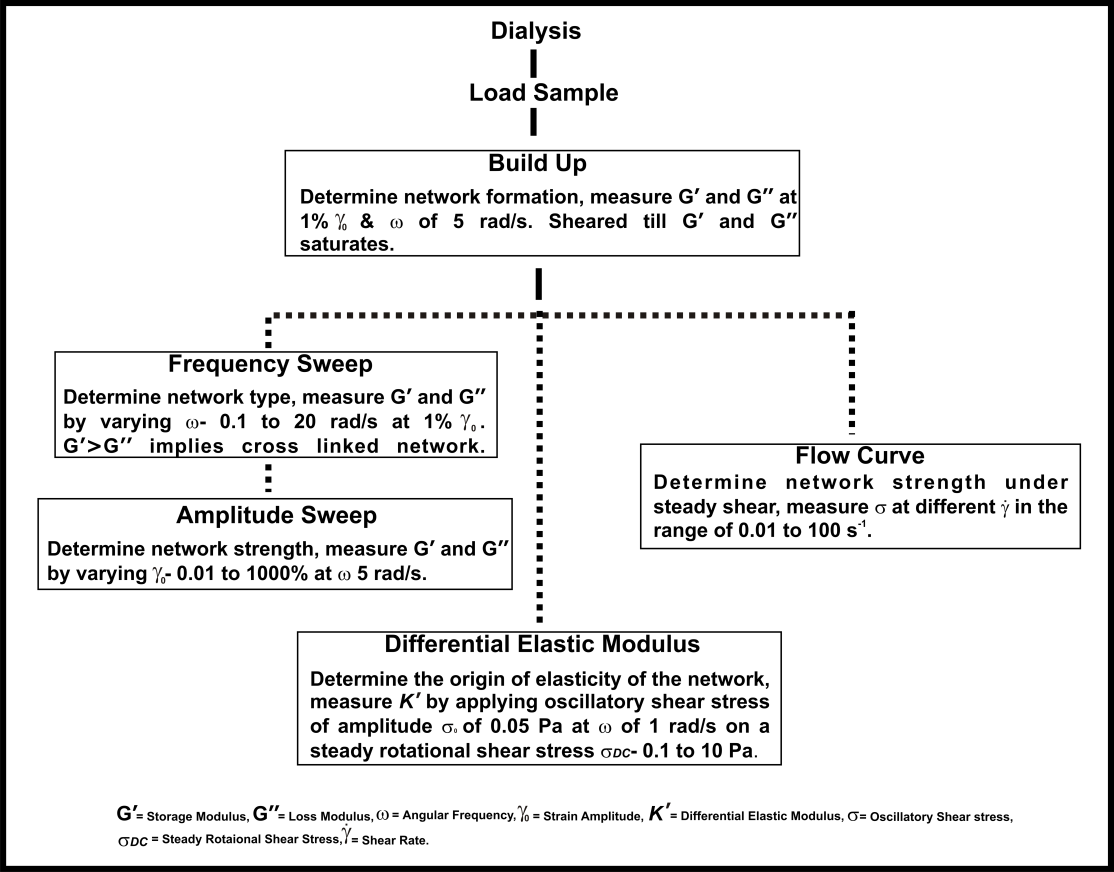
**

**Figure S1**: **Flow chart representing the scheme of the rheological measurements**.

**A**

**
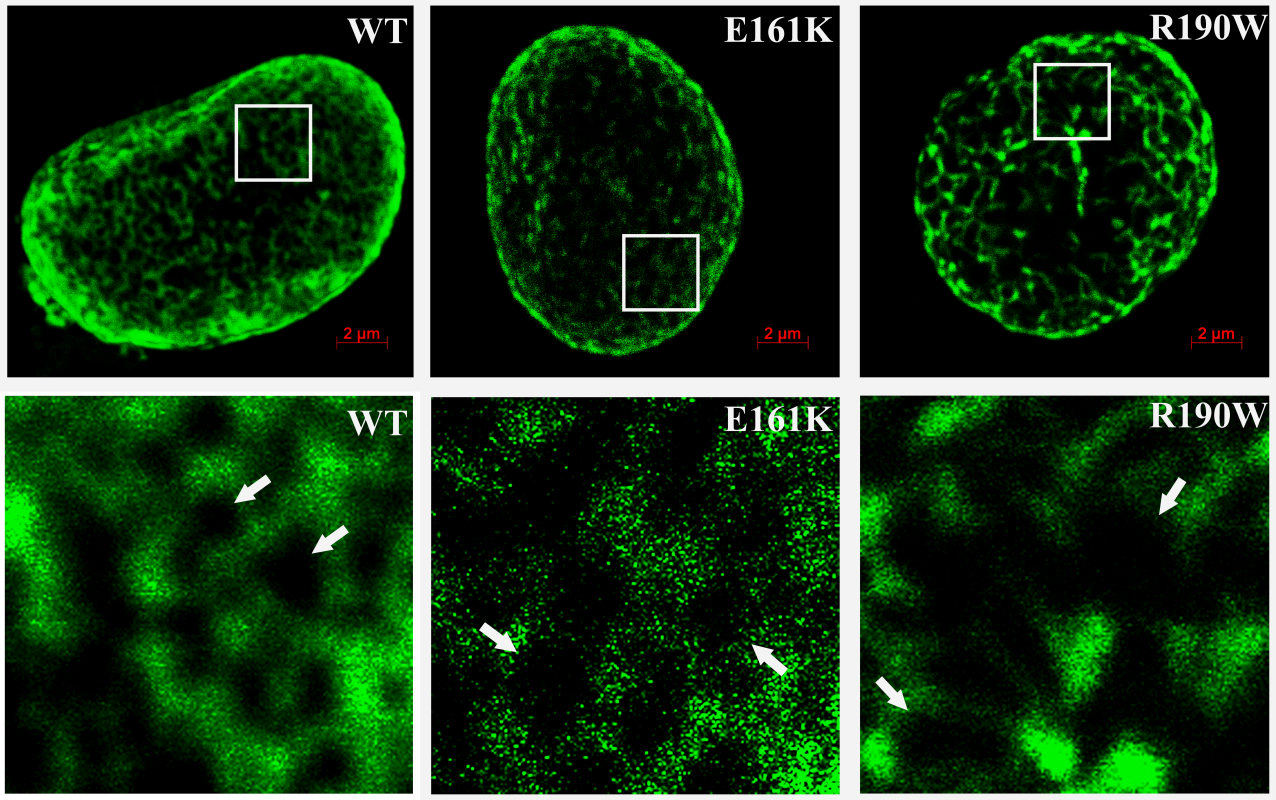
**

**B**

**
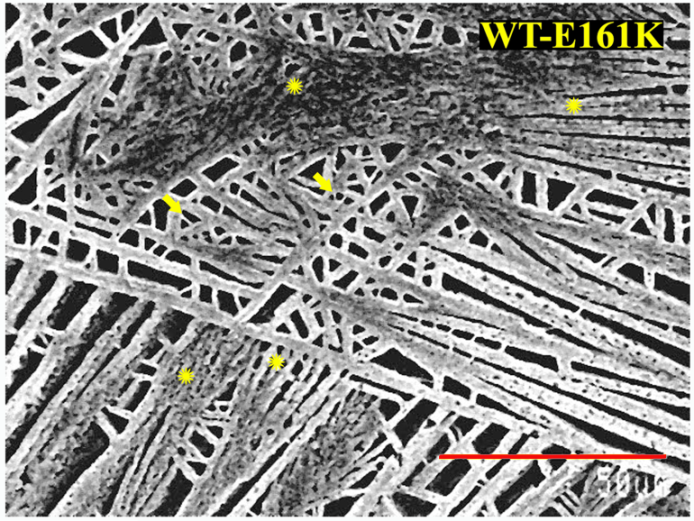
**

**Figure S2**: **Ultrastructure of lamin A network. A)** The nuclear lamina of HeLa cells transfected with pEGFP-LA and mutant constructs. Upper panels denote the wt LA, E161K and R190W respectively, whereas the bottom panels represent zoomed areas indicated by white squares which are magnified 7-fold in each case. The white arrows in the lower panels indicate the mesh area in the lamina. Scale bar = 2 μm. **B)** SEM image of hetero-polymerised wt LA and E161K network assembled *in vitro* in assembly buffer. Wild type and mutant protein were mixed in equal stoichiometry starting from 0.1 mg/ml up to a final concentration of 0.6 mg/ml. Images were acquired at a magnification 800x. Scale bar is 50 μm. Arrow and Asterisk marks indicate the cross-linked sites and bundled filaments in the network.

**
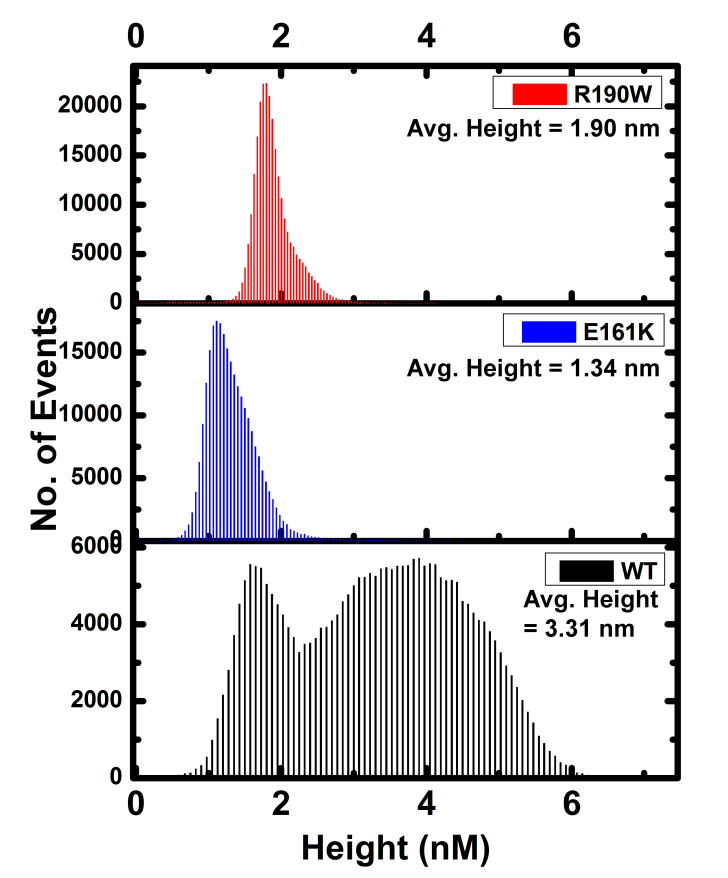
**

**Figure S3**: **Roughness profile of the *in vitro* assembled wild type and mutant proteins.** The surface roughness profiles of the network formed by wt LA, E161K and R190W, showed a filament height of ~3.31 nm, 1.34 nm and 1.90 nm for wt LA, E161K and R190W respectively.


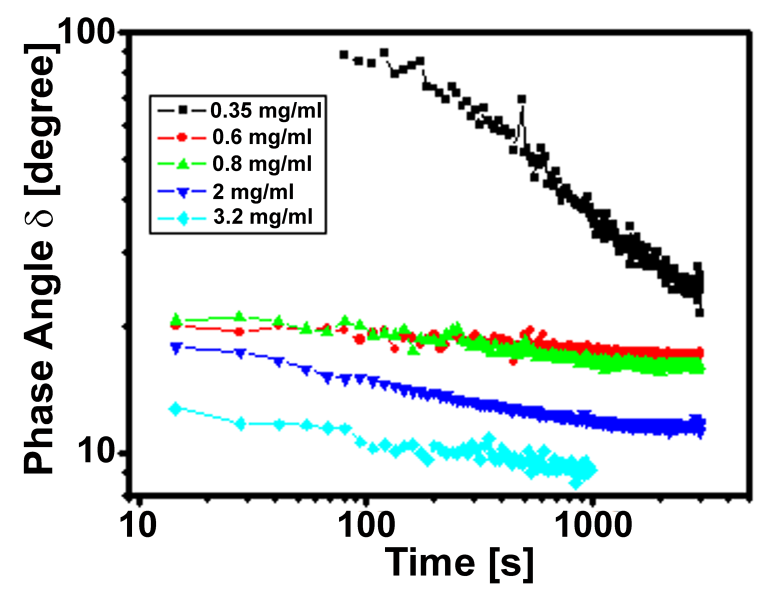


**Figure S4: Decrease in Phase angle δ with time.** Lamin A networks assembled *in vitro* at different concentrations exhibit a time dependent decrease in phase angle at constant frequency and amplitude under oscillatory shear.

**SUPPORTING INFORMATION REFERENCE**

1. Ando S, Nakao K-i, Gohara R, Takasaki Y, Suehiro K, et al. (2004) Morphological analysis of glutaraldehyde-fixed vimentin intermediate filaments and assembly-intermediates by atomic force microscopy. Biochim Biophys Acta 1702: 53-65.
